# Outcome of oligoprogressing metastatic renal cell carcinoma patients treated with locoregional therapy: a multicenter retrospective analysis

**DOI:** 10.18632/oncotarget.20022

**Published:** 2017-08-07

**Authors:** Daniele Santini, Raffaele Ratta, Francesco Pantano, Delia De Lisi, Marco Maruzzo, Luca Galli, Elisa Biasco, Azzurra Farnesi, Sebastiano Buti, Cora Nanette Sternberg, Linda Cerbone, Giuseppe Di Lorenzo, Silvia Spoto, Michelle Sterpi, Ugo De Giorgi, Rossana Berardi, Mariangela Torniai, Andrea Camerini, Francesco Massari, Giuseppe Procopio, Giuseppe Tonini

**Affiliations:** ^1^ Campus Bio-Medico University of Rome, Department of Medical Oncology, Rome, Italy; ^2^ Fondazione IRCCS, Istituto Nazionale dei Tumori, Oncology Unit 1, Milan, Italy; ^3^ Istituto Oncologico Veneto, IOV-IRCCS, Medical Oncology 1 Unit, Padova, Italy; ^4^ University Hospital of Pisa, Oncology Unit 2, Pisa, Italy; ^5^ University Hospital of Parma, Medical Oncology, Parma, Italy; ^6^ San Camillo and Forlanini Hospitals, Department of Medical Oncology, Rome, Italy; ^7^ Department of Clinical Medicine & Surgery, Oncology Division, University Federico II, Naples, Italy; ^8^ Campus Bio-Medico University of Rome, Department of Internal Medicine, Rome, Italy; ^9^ IRCCS Istituto Scientifico Romagnolo per lo studio e la Cura dei Tumori, Department of Medical Oncology, Meldola, Italy; ^10^ Università Politecnica delle Marche, Azienda Ospedaliero-Universitaria Ospedali Riuniti di Ancona, Medical Oncology Unit, Ancona, Italy; ^11^ Versilia Hospital and Istituto Toscano Tumori, Medical Oncology, Lido di Camaiore, Italy; ^12^ S.Orsola-Malpighi Hospital, Division of Oncology, Bologna, Italy

**Keywords:** metastatic renal cell carcinoma (mRCC), oligoprogression, pazopanib, sunitinib, targeted therapy

## Abstract

Locoregional treatment with radical intent should be considered during therapy with targeted agents in patients with metastatic renal cell carcinoma (mRCC) in order to achieve a complete response, especially in the setting of an oligo-progression in one or more metastatic sites.

We retrospectively enrolled 55 patients who experienced a disease oligo-progression after at least 6 months from the beginning of first-line therapy in one or more metastatic sites radically treated with locoregional treatments. Post-first-oligo-progression overall survival (PFOPOS) and post-first-oligo-progression free survival (PFOPFS) were evaluated.

The global median PFOPOS and PFOPFS were 37 months and 14 months respectively. Patients who continued the same therapy after a locoregional treatment on a site of progression had a significantly longer mPFOPOS compared to patients who changed therapy (39 *vs* 11 months, *p*=0.014). An advantage in mPFOPOS was also observed in patients with a Memorial Sloan-Kettering Cancer Center (MSKCC) good risk score compared to patients of the intermediate risk group (39 *vs* 29 months, *p*=0.036); patients with bone metastases had a longer mPFOPOS compared to those with visceral metastases (not reached *vs* 31 months, *p*=0.045). The only independent predictor of poor prognosis, in terms of PFOPOS at multivariate analysis (*p*=0.007), proved out to be change of treatment after first progression.

In this paper we aim to illustrate that continuing the same systemic therapy, after a radical locoregional treatment on a site of progression, seems to be associated with a prolongation of mPFOPOS.

## INTRODUCTION

In the last decade, the agents targeting the vascular endothelial growth factor receptor (VEGFR) (such as sunitinib, pazopanib, axitinib, sorafenib and bevacizumab) [1-6], the mammalian target of rapamycin (mTOR) pathway (including everolimus and temsirolimus) [7, 8] and the novel immune checkpoint-inhibitor antibody nivolumab [9] have revolutionized the treatment of metastatic renal cell carcinoma (mRCC) [10].

Nervertheless the introduction of targeted agents did not completely optimize treatment of mRCC because complete response (CR) is still rarely achieved. Recent findings seem to indicate that locoregional treatment such as surgery, radiotherapy or ablative techniques may improve survival in the metastatic setting, but only few retrospective analyses about their real impact on prolonging outcomes are available to date [11, 12].

In clinical practice locoregional treatment with radical intent should be considered during targeted therapy (TT) in order to attempt a CR, particularly in the context of a slow or oligo-progression in one or more metastatic sites; however currently no evidence on the correct TT sequencing when this progression occurs is available.

The concept of oligometastatic progression was first introduced by Hellman et al. [13, 14]. It can be considered as an intermediate biological state characterized by a restricted metastatic capacity and a transitional state prior to metastatic dissemination, with a limited number of sites of metastases involved. Therefore oligoprogression may be defined as a clinical situation where a limited number of metastatic tumor sites have progressed (usually between 3 and 5), while all other metastases are controlled by systemic therapy. In general it is possible for patients with oligometastatic progression, if approached with aggressive locoregional treatments, to achieve a satisfactory long-term survival not inferior to the one of non-metastatic patients’ [14].

Clinical data indicate that the number of renal cancer patients with oligometastatic disease receiving aggressive treatment is rapidly increasing [15]. In this subset of patients it remains unclear if it is more appropriate to continue the same TT or switch to another agent when progression occurs in one or more metastatic sites radically treated with a locoregional approach. Moreover no data about outcomes and no predictive and prognostic factors are defined in this specific population. In the present retrospective multicenter study, we reviewed the medical records of patients treated with first line TT who progressed after a long disease control in one or more metastatic sites radically treated with locoregional therapy. Outcome data were compared between patients who continued the same TT and patients who switched to another TT after oligo-disease progression. We also analyzed the predictive and prognostic impact of site of progression, type of locoregional treatment and switch to another TT on post-progression OS and PFS.

## PATIENTS AND METHODS

### Patients

This study enrolled 55 patients with advanced and/or metastatic RCC who had progressed, after a long lasting disease control, during first-line TT in one or more metastatic sites radically treated with locoregional therapy.

We retrospectively collected data from eleven Italian centers including patients treated form May 2007 to August 2015. June 2016 was the cut-off date of the last follow-up. Additional eligibility criteria consisted of a diagnosis of clear-cell or predominantly clear-cell histology; measurable disease per Response Evaluation Criteria In Solid Tumors (RECIST) criteria; age range from 18-75 years; an Eastern Cooperative Oncology Group (ECOG) performance status (PS) ≤ 2; a good or intermediate risk group according to the Memorial Sloan-Kettering Cancer Center (MSKCC) criteria (Motzer criteria) [16]; patients who progressed in maximum three metastatic sites radically treated with locoregional approach. Patients were excluded if they had an ECOG PS > 2, a poor MSKCC risk category and if they progressed within six months since the beginning of first-line TT. The locoregional treatments documented were surgery, radiotherapy or ablative techniques. The following data were recorded for each patient: date of nephrectomy, initial prognostic score based on Motzer criteria, date and site of oligo-progression, type of locoregional treatment, clinician’s choice of continuing or switching to another TT after progression radically treated with locoregional treatments, type of TT during the oligo progression.

### End points and assessment

The primary endpoint was post-first-oligo-progression-overall survival (PFOPOS), defined as the time interval between the date of first-oligo-progression radically treated with locoregional therapy and the date of death or of the last follow up. The main secondary endpoint was post-first-oligo-progression-free survival (PFOPFS), defined as the time interval between first radically treated oligo-progression in one or more metastatic sites and the second clinical or radiological progression. Type of locoregional treatment, site of progression and clinician’s choice of continuing or switching to another TT after locoregional progression were analyzed as predictive factors of PFOPFS and PFOPOS. In our analysis we prefer PFOPOS as primary endpoint since progression free survival is a surrogate of overall survival.

We also analyzed OS and PFS respectively defined as the time from the start of first line TT to death or of the last follow-up and the time from the start of first line TT to first clinical or radiological progression.

### Statistical analysis

OS, PFOPOS, PFS and PFOPFS were evaluated *via* the Kaplan-Meier method and Mantel-Haenszel log-rank test was employed to compare survival among groups. We considered distribution of clinical variables within each of the two groups of patients (continuing the same TT *vs* switch). Median OS (mOS), median PFOPOS (mPFOPOS), median PFS (mPFS) and median PFOPFS (mPFOPFS) were estimated by means of KaplanMeier product-limit method, while the Mantel-Haenszel log-rank test was used for statistical inference in the formal comparison between groups. A Cox-regression model was applied to the data with multivariate approach.

Multivariable logistic regression and propensity score analysis was performed in order to evaluate variables significantly associated with the continuation of the same TT after locoregional treatment of a site of oligoprogression. All significance levels were set at a 0.05 value. SPSS software (version 19.00, SPSS, Chicago) was used for all statistical analysis. Propensity score analysis was performed in R (v3.1.1) using nearest neighbor matching with R package “MatchIt”.

## RESULTS

### Patient characteristics

Fifty-five patients with advanced and/or mRCC have been identified from 11 Italian centers; all patients experienced a disease oligo-progression after at least 6 months from the start of first-line therapy in one or more metastatic sites that were radically treated with a locoregional therapy.

Most patients had clear-cell histology (94.5%, *N* = 52); 98.2% of patients (*N* = 54) had undergone prior nephrectomy (Table 1). According to the MSKCC classification, 56.4% of patients (*N* = 31) were good risk,

**Table 1:** Clinico-pathological characteristics of the overall population

Characteristics	No. of patients	%
**Gender**		
Male	43	78.2
Female	12	21.8
**ECOG PS**		
0-1	45	81.8
>1	10	18.2
**Previous nephrectomy**		
Yes No	54	98.2
	1	1.8
**Histology**		
Clear cell RCC	52	94.5
Non-clear cell RCC	3	5.5
**Sarcomatoid aspects**		
No	54	98.2
Yes	1	1.8
**Fuhrman grade**		
NA	10	18.2
G1	1	1.8
G2	25	45.5
G3	15	27.3
G4	4	7.3
**Metastasis at RCC diagnosis**		
No	36	65.5
Yes	19	34.5
**MSKCC**		
Good risk	31	56.4
Intermediate risk	23	41.8
N/A	1	1.8

PS = performance status; RCC = renal cell carcinoma; MSKCC = Memorial Sloan Kattering Cancer Center

41.8% of patients (*N* = 23) were intermediate risk and for 1 patient it was not possible to calculate the risk class; 65.5% of patients (*N* = 36) did not present metastases at diagnosis. The most common sites of oligo-progression were lung (*N* = 15) and bone (*N* = 10), followed by kidney (*N* = 8), brain and liver (*N* = 4 each). All patients collected presented oligo-progression in a single site. The majority of patients received sunitinib as first-line (87.3%, *N* = 48), followed by pazopanib (9.1%, *N* = 5) and sorafenib (3.6%, *N* = 2) (Table 2). 45.5% of patients (*N* = 25) received radiotherapy on the site of progression as locoregional therapy, 45.5% of patients (*N* = 25) underwent surgery and 9.1% (*N* = 5) were treated with ablative techniques (cryoablation or thermoablation). After radical treatment on progressing sites, 83.6% of patients (*N* = 46) did not switch to another tyrosine kinase inhibitor (TKI) and continued the same targeted agent used before the locoregional therapy; 12.8% of patients (*N* = 7) switched to another therapeutic agent (3 patients received an mTOR inhibitor [mTORi] and 4 were treated with a different TKI).

**Table 2:** Therapies, sites of oligo-progression and locoregional approaches

Characteristic	No. of patients	%
**First-line therapy**		
Sunitinib	48	87.3
Pazopanib	5	9.1
Sorafenib	2	3.6
**Site of oligo progression**		
Bone	10	18.2
Brain	4	7.3
Controlateral kidney	8	14.5
Liver	4	7.3
Lung	15	27.3
Others	14	25.4
**Type of locoregional treatment**		
Ablative techniques	5	9.1
Surgery	25	45.5
Radiotherapy	25	45.5
**Therapy after first progression**		
NA	1	1.8
Change (mTORi, TKI)	7	12.8
No change	46	83.6
No treatment	1	1.8

mTORi = mammalian target of rapamicyn inhibitor; TKI= tyrosine kinase inhibitor

### Outcome analyses: post-first-oligo-progression overall survival

We evaluated mOS from disease progression in one or more sites treated with locoregional approaches, that we defined as post-first-oligo-progression overall survival (PFOPOS): mPFOPOS was 37 months (95% Confidence Interval [CI] 25.2-48.7).

**Figure 1:**
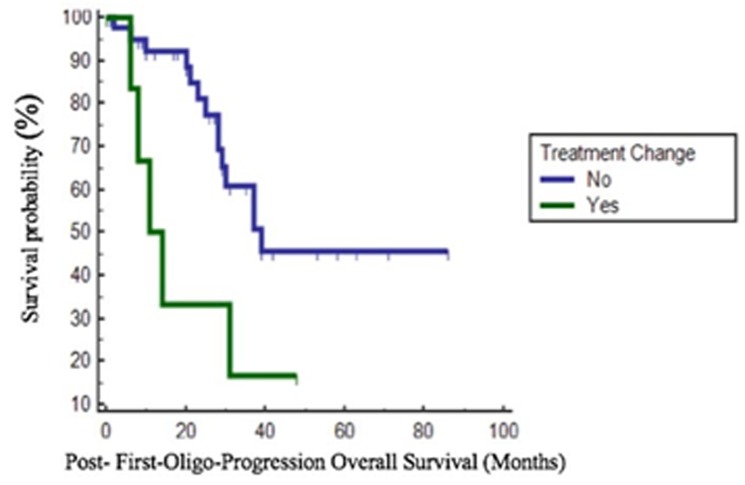
Post-First-Oligo-Progression Overall Survival (PFOPOS) according to treatment change after first oligo progression

We further analyzed PFOPOS based risk class: patients with a good risk score had a longer mPFOPOS compared to patients of the intermediate risk group (39 *vs* 29 months, *p* = 0.036). Patients who developed bone metastases as site of oligo-progression had a trend of longer mPFOPOS compared to those who developed visceral metastases (not reached [NR] *vs* 31 months, *p* = 0.045). Patients that received the same therapy after a locoregional treatment on progressing sites had a longer mPFOPOS compared to patients who switched to another therapy (39 *vs* 11 months, *p* = 0.014) (Figure 1). Patients with Fuhrman grade 1 and 2 presented a better mPFOPOS than patients with Fuhrman grade 3 and 4 (57 *vs* 37 months, *p* = 0.021).

None of the other variables that have been considered (such as age, gender, ECOG PS, histology and type of TKI received) were significantly associated with a prolongation in survival after the oligo-progression (Table 3). At multivariate Cox regression analysis, only change of treatment after first progression (*p* = 0.007, HR 6.280) was an independent predictor of poorer prognosis in terms of PFOPOS (Table 4).

**Table 3:** PFOPOS in patients with renal cell carcinoma after locoregional approach on oligo-progressing sites

	PFOPOS (months)	p-value
**Age**		0.146
< 58 y	NR	
≥ 58 y	30	
**Gender**		0.639
Male	NR	
Female	37	
**ECOG PS**		0.641
0-1	37	
<1	NR	
**Histology**		0.941
Clear cell RCC	37	
Non-clear cell RCC	NR	
**Fuhrman grade**		**0.021**
G1 + G2	57	
G3 + G4	37	
**MSKCC**		**0.036**
Good risk	39	
Intermediate risk	29	
**First-line therapy**		0.380
Sunitinib	42	
Pazopanib	11	
Sorafenib	2	
**Site of first progression**		**0.045**
Bone	NR	
Visceral	31	
**Type of locoregional treatment**		0.558
Ablative techniques	NR	
Surgery	NR	
Radiotherapy	37
**Site of progression**		0.504
Bone	58	
Brain	25	
Controlateral kidney	29	
Liver	22	
Lung	37	
Others	37	
**Therapy after first progression**		**0.014**
Change (mTORi, TKI) 39 No change	11	

CI= confidence interval; PS = performance status; RCC = renal cell carcinoma; MSKCC = memorial sloan kattering cancer center; mTORi = mammalian target of rapamicyn inhibitor; TKI= tyrosine kinase inhibitor; NR = not reached.

**Table 4:** Multivariate analyses of predictors of PFOPOS

PFOPOS	Multivariate Cox regression	
	HR	*p-value*
**MSKCC** Good risk vs Intermediate risk	1.747	0.337
**Site of first progression** Bone vs Visceral	3.772	0.231
**Therapy after first progression** Change vs No change	**6.280**	**0.007**
**Fuhrman grade** G1+G2 vs G3+G4	1.156	0.835

Logistic regression analysis showed that brain metastases and radiotherapy at oligo-progression site were significantly associated with continuing the same TT (Supplementary Table S1 on Supplementary Materials).

Anyway in a subsequent propensity score analysis only brain metastases demonstrated to have an influence on the decision to continue the same TT (Supplementary Table 2 on Supplementary Materials).

### Outcome analyses: post-first-oligo-progression-free survival

**Figure 2:**
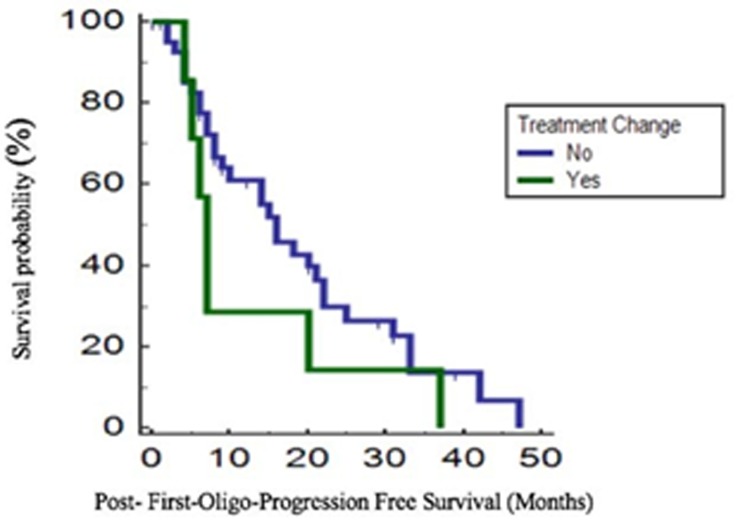
Post-First-Oligo-Progression Free Survival (PFOPFS) according to change of treatment after first progression

We also evaluated post-first-oligo-progression-free survival (PFOPFS), defined as the time interval between disease oligo-progression in one or more metastatic sites, treated with locoregional approaches, and the first subsequent clinical or radiological progression. mPFOPFS in all population was 14 months (95% CI 6.9-21). None of the variables considered (ECOG PS, age, gender, histology, Fuhrman grade, prior nephrectomy, risk group and therapy received) were significantly associated with a prolongation in PFOPFS after the locoregional treatment on oligo-progressing sites. Multivariate analysis failed to show any independent prognostic factor in terms of PFOPFS. No statistically significant difference in terms of mPFOPFS was found between patients who continued the same treatment after disease oligo-progression and those who changed therapy (15 *vs* 7 months, *p* = 0.207) with a trend in favour of patients who did not switch therapy (Figure 2).

We also compared median global PFS (defined as the sum of PFS at the primary oligo-progression plus the PFOPFS) and no statistically significant difference was found between patients who received the same treatment after oligo-progression and those who underwent a change in therapy (43 *vs* 34 months respectively, *p* = 0.339), with a trend in favour of the group continuing with the same agent.

## DISCUSSION

In the last decade, the agents targeting the VEGFR, the mTOR pathway and the novel immune checkpointinhibitor antibody nivolumab have revolutionized the treatment of mRCC [17-20]. The majority of patients treated with targeted therapy achieve a stable disease (SD) or a partial response (PR) according to RECIST criteria as their best response, furthermore a subset of patients with favorable risk factors have a long lasting duration of response during treatment and a long survival (more than 30 months) [21, 22].

The role of locoregional treatments in mRCC has played an important role in tumor control even though only few retrospective analyses about their real impact on prolonging outcomes are available to date [11].

A matter of debate remains whether TT should be continued after CR. In 2012, Albiges and her group presented the results of a multicenter retrospective analysis including 64 patients who achieved complete response (CR) during treatment with sunitinib or sorafenib administered alone or with local approaches [23]. In this study, 53 patients (83%) stopped treatment after CR: of them, 29 patients (17 who had obtained CR with TT alone and 12 with additional local treatments) were still in CR at time of analysis, suggesting the possibility of a drug holiday after achieving CR. Data about this subset of patients were also reviewed by our group in a multicenter study that retrospectively analyzed the risk of recurrence and conditional Disease-Free Survival (cDFS) in 63 patients with complete remission during treatment with TKI, alone or with local treatment in mRCC [24]. On the basis of these data we found that patients who obtained CR with the help of multimodal approaches presented lower rate of recurrence (40% *vs* 61%) and longer DFS compared to patients

treated with TKI alone (16.5 *vs* 41.9 months, *p* = 0.039). Moreover the rate of recurrence was higher in patients with brain (88%), pancreatic (71%) and bone metastases (50%). As far as DFS, patients who continued TKI therapy after a complete response had a longer DFS than those who stopped therapy, although the difference was not statistically significant (42.1 *vs* 25.1 months, *p* = 0.254). Additionally two years cDFS was better in patients who were treated with multimodal treatment and continued TKIs than in the other arm of patients. In the last years, the use of locoregional therapy has increased in order to manage oligo-progressing disease and attempt a complete response. To date, no data about the correct strategy after oligo-progression are available.

In this study we retrospectively investigated the real world therapeutic strategy for patients with mRCC who experienced a disease oligo-progression after at least 6 months from the start of first-line therapy in one or more metastatic sites and were treated with a locoregional approach (surgery, radiotherapy or ablative tecniques) with radical intent.

Considering the PFPOS as the primary endpoint of our analysis, we showed that continuing the same therapy after a locoregional treatment on a site of progression seems to be associated with a prolongation of median post-progression overall survival. In addition, we pointed out that patients who developed bone metastases as site of oligo-progression had a trend of longer post progression mOS compared to those who developed visceral metastases. In this regard, there are opposing literature data: some studies suggest that bone metastases have a negative impact and are associated with poor clinical outcomes in patients with mRCC [25-27]. Others show that RCC patients with less than 5 bone metastases have favorable outcomes under therapy with sunitinib [28]. This could be related to a less biological aggressiveness of progression in bone metastases compared to a progression in visceral metastases [29].

Furthermore several retrospective analyses showed that patients with single or oligo-metastatic RCC bone disease who underwent locoregional treatment presented a better overall survival and tumor control than patients with both bone and visceral metastases locally treated [11, 30, 31].

No other variables were clearly associated with a prolonged OS, except for patients with good Fuhrman grade, confirming the prognostic value of nuclear grade in RCC [32]. Thus our data seem to suggest that the continuation of the same TKI after oligo-progression represents the best clinical option.

Considering all baseline variables, we found that radiotherapy and brain metastases were significantly associated with continuing the same TT.

This may be explained by the fact that TKI and mTOR inhibitors rarely cross the blood-brain barrier, so when only brain progression occurs TT combined with radiation therapy can be safely carried out in this subset of RCC patients [33].

On the other hand, even though there was no statistically significant difference in terms of PFOPFS between patients that received the same treatment after disease oligo-progression and those that changed therapy, we observed a trend in favour of the group that continued with the same regimen. These patients recorded 15 months of additional PFS after the first oligo-progression. Furthermore, considering global PFS (defined as the sum of PFS at the primary oligo-progression plus PFOPFS), we found no statistically significant difference between patients, with a trend in favour of those who continued the same TT, suggesting that the switch to a second TT should be strongly delayed in this subset of patients.

Althought the eterogeneity and the small sample size analized, our data suggest that continuing the same TKI after oligoprogression prolong overall survival, allowing clinicians to have further therapeutic choices in case of progression.

We could probably explain the difference between the two end- point considered, supposing that during TKI therapy resistant tumor cells are selected causing oligroprogression, so when these sites are radically treated only tumor cells who respond to TKI remains.

Even if there are no published guidelines regarding this topic, our findings are in line with current clinical practice. Indeed, most of the patients in our records continued the same therapeutic agent after locoregional treatment on oligo-progressing sites.

The compelling finding in our study is that using the same agent, until a clear progression occurs, allows to delay the use of a different drug, therefore saving a convenient therapeutic option for later use.

However, there are several limitations to this study. First off its retrospective design increases the risk of incurring bias in data selection and analysis. Secondly, the small number of patients included might give a distorted estimate of patients’ characteristics. Thirdly, there is the discrepancy in the number of enrolled patients who continued the same therapy *versus* those who changed agent, seemingly a result, as mentioned before, of the current clinical practice. An interesting aspect that could be studied in a successive analysis is the evaluation of the site of progression after locoregional treatment, in order to assess if progression occurs at the same site or not. According to an exploratory analysis in a subset of patients included in the present study, it seems that the subsequent progression occurs in a different site from the first oligo-progression.

Despite these limitations, the present study is the first retrospective analysis to evaluate outcome in this specific good prognostic subset of patients, suggesting that manteining the same drug after locoregional treatment on oligo-progressing sites should be carefully considered in RCC patients.

Considering the selective population and the differences between locoregional approaches in the institutions involved, only a retrospective analysis could be considered. Nonetheless, perspective studies are needed to identify and validate specific predictive factors associated with outcomes in this population, including standardization of the locoregional techniques for the treatment of the oligo-progression.

## SUPPLEMENTARY MATERIALS TABLES


