# Supplementary material for: Outcome of oligoprogressing metastatic renal cell carcinoma patients treated with locoregional therapy: a multicenter retrospective analysis
Source: Oncotarget. 2017 Aug 7;8(59):100708–16. doi: 10.18632/oncotarget.20022 (PMC5725056; doi:10.18632/oncotarget.20022)
Supplement: Supplementary file 1 [file oncotarget-08-100708-s001.pdf]

Outcome of oligoprogressing metastatic renal cell carcinoma patients treated with locoregional therapy: a multicenter retrospective 3 analysis

SUPPLEMENTARY MATERIALS

Supplementary Table 1: Logistic regression analysis

		Score	df	Sig.
Variables	Sex	,428	1	,513
	ECOG	,212	2	,899
	ECOG(1)	,131	1	,717
	ECOG(2)	,137	1	,711
	Nefrectomy	,266	1	,606
	Hystology	,695	1	,405
	Fhurman Grade	,502	4	,973
	Fhurman Grade 1	,137	1	,711
	Fhurman Grade 2	,131	1	,717
	Fhurman Grade 3	,057	1	,811
	Fhurman Grade 4	,242	1	,623
	Metastatic at diagnosis	,041	1	,839
	Brain	,547	1	,459
	Bone	2,897	1	,089
	Kidney	,682	1	,409
	Lymphnode	2,846	1	,092
	Pancreas	1,333	1	,248
	Liver	,010	1	,919
	Lung	1,562	1	,211
	Motzer risk	,148	2	,929
	Motzer risk 1	,131	1	,717
	Motzer risk 2	,007	1	,932
	TKI 1 line	1,784	2	,410
	TKI 1 line 1	1,165	1	,280
	TKI 1 line 2	,772	1	,380
	Logoregional Therapy	7,394	2	,025
	Logoregional Therapy 1	,847	1	,357
	Logoregional Therapy 2	4,705	1	,030
	Site of progression	10,726	6	,097
	Bone	2,676	1	,102

	Brain	6,065	1	,014
	Kidney	,010	1	,919
	Liver	,772	1	,380
	Lung	,657	1	,418
	Node	,404	1	,525

Supplementary Table 2: Propensity score analysis

Coefficients:

	Estimate	Std,.Error	t	p.value
(Intercept)	-0,262125	0,618778	-0,424	0,6739
Age	0,002937	0,004724	0,622	0,5374
Sex	0,10559	0,117595	0,898	0,374
ECOG0-1	0,073168	0,128311	0,57	0,5714
Nefrectomy Yes	-0,073096	0,445656	-0,164	0,8705
Hystology Not Clear Cell	-0,009347	0,309474	-0,03	0,976
Metastatic at Diagnosis	-0,026821	0,105198	-0,255	0,7999
Brain	-0,468683	0,231826	-2.022	0,0492
Bone	-0,05305	0,172346	-0,308	0,7596
Kydney	-0,023322	0,141439	-0,165	0,8698
Node	-0,045677	0,112861	-0,405	0,6876
Pancreas	-0,009729	0,152859	-0,064	0,9495
Liver	0,120254	0,293012	0,41	0,6835
Lung	0,013677	0,11789	0,116	0,9082
Motzer Score	-0,059968	0,118925	-0,504	0,6165
First Line TKI	0,002903	0,087174	0,033	0,9736
Progression Site Brain	0,449739	0,251037	1.792	0,0799
Progression Site Kidney	0,164239	0,218269	0,752	0,4557
Progression Site Liver	0,011841	0,366962	0,032	0,9744
Progression Site Lung	0,038207	0,197668	0,193	0,8476
Progression Site Node	0,099033	0,258259	0,383	0,7032
Progression Site Other	-0,044049	0,178441	-0,247	0,8061
Locoregional Treatment Radiotherapy	0,004948	0,214104	0,023	0,9817
Locoregional Treatment Surgery	0,161575	0,204959	0,788	0,4346
